# Single-Strand Conformation Polymorphism Fingerprint Method for Dictyostelids

**DOI:** 10.3389/fmicb.2021.708685

**Published:** 2021-08-27

**Authors:** Phongthana Pasookhush, Asmatullah Usmani, Kowit Suwannahong, Prasit Palittapongarnpim, Kamolchanok Rukseree, Kanchiyaphat Ariyachaokun, Sureemas Buates, Suradej Siripattanapipong, Pravech Ajawatanawong

**Affiliations:** ^1^Division of Bioinformatics and Data Management for Research, Research Division, Faculty of Medicine Siriraj Hospital, Mahidol University, Bangkok, Thailand; ^2^Department of Microbiology, Faculty of Science, Mahidol University, Bangkok, Thailand; ^3^Department of Biology, Faculty of Education, Kandahar University, Kandahar, Afghanistan; ^4^Department of Environmental Health, Faculty of Public Health, Burapa University, Chonburi, Thailand; ^5^National Science and Technology Development Agency (NSTDA), Thailand Science Park, Khlong Nueng, Thailand; ^6^Department of Sciences and Liberal Arts, Mahidol University, Amnatcharoen Campus, Bung, Thailand; ^7^Department of Biological Sciences, Faculty of Science, Ubon Ratchathani University, Ubon Ratchathani, Thailand

**Keywords:** SSCP, dictyostelids, fingerprint, *SSU rDNA* gene, diversity

## Abstract

Dictyostelid social amoebae are a highly diverse group of eukaryotic soil microbes that are valuable resources for biological research. Genetic diversity study of these organisms solely relies on molecular phylogenetics of the *SSU rDNA* gene, which is not ideal for large-scale genetic diversity study. Here, we designed a set of PCR–single-strand conformation polymorphism (SSCP) primers and optimized the SSCP fingerprint method for the screening of dictyostelids. The optimized SSCP condition required gel purification of the SSCP amplicons followed by electrophoresis using a 9% polyacrylamide gel under 4°C. We also tested the optimized SSCP procedure with 73 Thai isolates of dictyostelid that had the *SSU rDNA* gene sequences published. The SSCP fingerprint patterns were related to the genus-level taxonomy of dictyostelids, but the fingerprint dendrogram did not reflect the deep phylogeny. This method is rapid, cost-effective, and suitable for large-scale sample screening as compared with the phylogenetic analysis of the *SSU rDNA* gene sequences.

## Introduction

Dictyostelid social amoebae are a group of non-pathogenic, unicellular eukaryotic microbes commonly found in diverse ecosystems throughout the world (Baldauf and Strassmann, 2017). They are important eukaryotic microbes in soils that control the bacterial population and play a role in nutrient recycling (Cavender and Raper, 1965). They generally live as unicellular amoebae. When food is depleted, the amoeba can aggregate and together differentiate into a multicellular slug-like organism that eventually transforms into a multicellular fruiting body. So far, more than 150 species have been discovered and described (Baldauf et al., 2018). Of the known dictyostelids, *Dictyostelium discoideum* is the only well-studied species and is used as a model microorganism for various biological processes, including autophagy (Calvo-Garrido et al., 2010; Mesquita et al., 2017), phagocytosis (Vogel et al., 1980; Bozzaro and Eichinger, 2011), cell signaling (Devreotes, 1989), and chemotaxis (Gerisch, 1982; King and Insall, 2009).

The first species was described in the 1800s; since then, they have been continuously discovered especially from nutrient-rich habitats, which were believed to have large species diversity (Baldauf et al., 2018). However, more recently, soil samples from poor habitats have also shown a large diversity of dictyostelids (Landolt et al., 2006). The molecular phylogeny was shown to be a more reliable indicator for the systematics of dictyostelid social amoebae rather than the traditional morphology-based approach (Schaap et al., 2006). The subsequent study of systematic phylogenies of dictyostelids has redefined the classification from eight major groups (Romeralo et al., 2011, 2012) to 12 genera (Sheikh et al., 2018). Analysis of molecular diversity by culture-independent environmental DNA (eDNA) sampling of *SSU rDNA* gene revealed the high diversity of uncultured dictyostelids and suggested that the true diversity has not yet been discovered (Baldauf et al., 2018).

The *SSU rDNA* gene is a standard biomarker for the classification of eukaryotic organisms, including dictyostelids, with combined phylogenetic analysis. This marker is also commonly used for known dictyostelids (Sheikh et al., 2018). However, molecular phylogenetic-based classification could be very costly for a larger sample size. Despite that the genus-level classification of dictyostelids using the 5′-end of the *SSU rDNA* gene helps to reduce the DNA sequencing reaction costs by half (Rukseree et al., 2018), the cost of DNA sequencing is still high for large-scale diversity surveys. Given the expected large numbers of undiscovered species and the potential utility of these microorganisms in biological research, a rapid and cost-effective method for large-scale screening would be helpful in understanding the extent and general taxonomic distribution of this hidden diversity.

Single-strand conformation polymorphism (SSCP) is a fingerprint technique whereby the migration patterns are determined by the secondary structures of single-stranded DNA (ssDNA) molecules, resulting in sequence-specific fingerprints (Olive and Bean, 1999; Hashim and Al-Shuhaib, 2019). The SSCP method requires four steps: (i) polymerase chain reaction (PCR) amplification of a short DNA fragment, (ii) denaturation of the PCR amplicons to generate the ssDNA, (iii) rapid decrease of the temperature to allow the ssDNA to form unique single-strand conformations without reannealing to the complementary strand, and (iv) detection of the ssDNA conformations by electrophoresis (Orita et al., 1989; Hayashi, 1991, 1992). The SSCP fingerprint method can detect more than 90% of a single mutation in fragments of less than 200 nucleotides and more than 80% in 300–400 nucleotide fragments (Hayashi, 1991, 1992; Hayashi and Yandell, 1993). Taken together, with the combination of highly sensitive DNA staining methods such as silver staining (Bassam and Gresshoff, 2007), the SSCP technique was applied to genetic diversity studies of bacteria and fungi. These studies generally used conserved DNA fragments such as the *ribosomal RNA (rDNA)* gene, the internal transcribed spacer (ITS), *16S rDNA gene*, and *18S rDNA gene* (Hayashi, 1992; Hauser et al., 2001; Stach et al., 2001; Abu et al., 2011).

In this study, we aimed to optimize the SSCP fingerprint method for the diversity study of dictyostelids. We began by designing a new set of universal primers within the *SSU rDNA* gene that could represent the genetic diversity of dictyostelids by the SSCP method. Once the SSCP fingerprint method was optimized, the 73 Thai isolates of dictyostelids that were isolated previously (Rukseree et al., 2018) were used to validate this SSCP fingerprint method.

## Materials and Methods

### Dictyostelids Samples and DNA Extraction

A total of 73 Thai isolates of dictyostelids used in this study were isolated from the soil samples collected from Phana and Mueang districts, Amnat Charoen province, Thailand, in December 2014 and July 2015 (Rukseree et al., 2018). These isolates were phylogenetically assigned as five genera, including the genus *Cavenderia* (17 isolates), *Polysphondylium* (15 isolates), *Dictyostelium* (35 isolates), *Raperostelium* (five isolates), and a single isolate of the genus *Heterostelium*. All dictyostelid isolates were cultured on non-nutrient agar (NNA) spread with *Escherichia coli* ATCC8739 as described previously (Rukseree et al., 2018).

To extract the DNA of dictyostelids, the amoeboid cells in the clear zone on the NNA plates were harvested and suspended in 200 μl of Tris-EDTA (TE) buffer. The cell suspension was then subjected to genomic DNA extraction using AxyPrep Blood Genomic DNA Miniprep Kit (Axygen Biosciences, Union City, CA, United States) according to the manufacturer’s protocol.

### Design of Single-Strand Conformation Polymorphism Primers

The SSCP primer design started with the identification of highly conserved regions in the target sequence. So a total of 192 *SSU rDNA* gene sequences were downloaded from the GenBank database (Altschul et al., 1990). Of those 192 sequences, nine of them were Thai strains isolated from Amnat Charoen province, Thailand (Rukseree et al., 2018); and the other 183 sequences were selected from the *SSU rDNA* gene dataset to represent dictyostelid diversity (Sheikh et al., 2018). The entire 192 *SSU rDNA* gene sequences belonged to 12 genera of dictyostelids, including 11 isolates of *Acytostelium* spp., 31 isolates of *Cavenderia* spp., four isolates of *Coremiostelium* spp., 56 isolates of *Dictyostelium* spp., five isolates of *Hagiwaraea* spp., 44 isolates of *Heterostelium* spp., eight isolates of *Polysphondylium* spp., 22 isolates of *Raperostelium* spp., *Rostrostelium ellipticum* AE2, *Speleostelium caveatum* WS695, two isolates of *Synstelium* spp., and seven isolates of *Tieghemostelium* spp. (Supplementary Table 1). All the sequences were aligned using the MUSCLE alignment program (Edgar, 2004) via SeaView version 5.0.4 (Gouy et al., 2010). Shannon’s entropy and the sequence similarity scores of each alignment position were calculated along the multiple sequence alignment (MSA) (Schmitt and Herzel, 1997; Shannon, 1997). Shannon’s entropy of the alignment position *i*^th^ (*S*_*j*_) was calculated by

$$S_{i}=-\sum_{m=1}^{n}\left(P\,\left(x_{m}\right)\,l\,o\,g\,P\,\left(x_{m}\right)\right)$$

where *P*(*x*_*m*_) is the probability of each nucleotide in position *m*, and the total number of nucleotide states in each alignment position equals five (A, T, C, G, and –). The sequence similarity scores were calculated using the percentage of the most frequent nucleotide in each position.

The PCR-SSCP primers were designed based on three criteria. First, the primers must be universal, which can bind to all *SSU rDNA* sequences of dictyostelids. Second, the expected PCR amplicons must be approximately 200 base pairs. Third, the primer binding sites should be located in regions with low entropy and high similarity. Melting temperatures and secondary structure predictions, including self-dimer, hairpin, and heterodimer, were calculated using the OligoAnalyzer^TM^ tool^1^. The specificity of the primers was validated using two blast software. Both forward and reverse primers were used as input to NCBI’s Primer-BLAST, with the default parameters (Ye et al., 2012). Each primer was also used as an input in NCBI’s BLASTn with the parameters automatically adjusted for a short query sequence (Altschul et al., 1990).

### PCR–Single-Strand Conformation Polymorphism of the Partial SSU rDNA Fragment and Amplicon Purification

The partial *SSU rDNA* gene was amplified using the forward primer (SSCP6_F: 5′-GCAGTAAATCGGGGCTAATAC-3′) and the reverse primer (SSCP6_R: 5′-CCCGTTACAACCATGGTA-3′). The PCR consisted of 0.2 μM of each primer, 1.5 mM of MgCl_2_, 200 μM of each dNTP, 1X of PCR Reaction Buffer, 1.25 U of *Taq* DNA polymerase (biotechrabbit, Berlin, Germany), and 12 ng of genomic DNA template. The final volume of the reaction was adjusted to 50 μl with sterilized deionized water. The PCR condition was initial denaturation at 95°C for 5 min, followed by 30 cycles of 95°C for 30 s, 55°C for 30 s, 72°C for 30 s, and the final extension at 72°C for 5 min. The amplicons were analyzed using 3% agarose gel electrophoresis followed by SERVA DNA STAIN G staining (SERVA, Heidelberg, Germany).

The PCR amplicons were purified using the FavorPrep^TM^ GEL/PCR Purification kit (Favorgen Biotech, Pingtung, Taiwan), according to the manufacturer’s protocol. The purification process was performed under two conditions: (i) direct purification from PCR mixtures and (ii) purification from the agarose gels. Unpurified PCR amplicons and purified PCR amplicons in both conditions were later subjected to the SSCP fingerprint method.

### Single-Strand Conformation Polymorphism Fingerprint Method

The SSCP fingerprint method was started by mixing 7 μl of the PCR amplicons with 7 μl of loading buffer containing 96% v/v formamide (1:1 ratio). The mixture was heated at 95°C for 10 min for denaturation of the double-stranded DNA (dsDNA) and then immediately chilled on ice to allow the ssDNA to rapidly fold into a unique conformation and maintain the ssDNA conformation. Then, 5 μl of the denatured amplicons was loaded into a freshly prepared 9% non-denatured polyacrylamide gel (9% acrylamide, 0.25% glycerol, 50 mM of Tris–HCl, 50 mM of boric acid, and 1 mM of EDTA) at 4°C. The 100-bp DNA ladder (New England Biolabs, Ipswich, MA, United States) was loaded into the first and last bands of each gel as a reference marker. Non-denatured polyacrylamide gel electrophoresis (non-denatured PAGE) was performed at 130 V, 4°C for 3 h using Bio-Rad Mini-PROTEAN 3 Cell Electrophoresis System (Bio-Rad Laboratories, Hercules, CA, United States). The electrophoresis tank was filled with 0.5X TBE buffer (50 mM of Tris–HCl, 50 mM of boric acid, and 1 mM of EDTA). After electrophoresis, the polyacrylamide gel was subjected to silver staining (Bassam and Gresshoff, 2007) and documented with the ChemiDoc^TM^ XRS+ system (Bio-Rad Laboratories, Hercules, CA, United States).

### Bioinformatics Analyses

All 73 SSCP fingerprints of the dictyostelid Thai isolates were recorded in the TIFF file format. The fingerprint analysis was conducted using GelJ software version 2.0 (Heras et al., 2015). Briefly, each gel’s lanes were manually detected using the rectangle tool with arcs to account for the gel smiling effect. Each band of the fingerprint was then manually identified by the peak of the band intensity histogram. For the comparison between different gels, normalization of each gel was performed individually using a standard curve generated by the software. The standard curve of each gel was generated using the migration rate of each band of reference marker with a Gaussian model of migration (*R*^2^ ≥ 0.95) (Heras et al., 2015). For clustering analysis of the SSCP patterns, an SSCP dendrogram was constructed from a similarity matrix of the fingerprint using the band difference similarity method with a tolerance parameter of 4. The similarity between two fingerprints was calculated based on the number of matching/non-matching bands at a given tolerance as described by Heras and others (Heras et al., 2015).

The partial sequences of the *SSU rDNA* gene of 73 dictyostelid Thai isolates (Rukseree et al., 2018) were downloaded from the GenBank database (Altschul et al., 1990). The sequences were aligned using the MUSCLE program (Edgar, 2004) through SeaView version 5.0.4 (Gouy et al., 2010). The MSA was manually edited, with the incomplete sequences at both ends of the MSA being truncated. Two maximum likelihood (ML) phylogenies were reconstructed from the full *SSU rDNA* gene and ∼200 bp of the PCR-SSCP amplicons using RAxML program version 8.2.12 (Stamatakis, 2014). A total of 1,844 and 232 aligned positions were used for the phylogenetic reconstruction of the full *SSU rDNA* gene and the PCR-SSCP amplicons, respectively. The tree was built using the GTR model with some optimization of substitution rates, GAMMA model of rate heterogeneity, and an estimated proportion of invariable sites (-m GTRGAMMAI). The bootstrap values were calculated from 1,000 replicates.

The prediction of the RNA secondary structures was calculated by the RNAfold WebServer^2^ within ViennaRNA Package 2.0 (Lorenz et al., 2011). The program parameters were set as default, and the results were presented in minimum free energy (MFE) structures.

## Results

### Primers for Single-Strand Conformation Polymorphism Fingerprint Method for Dictyostelids

**FIGURE 1:**
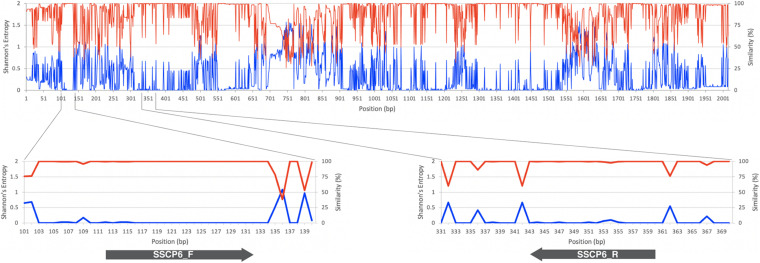
*SSU rRNA* gene sequence conservation and positions of the single-strand conformation polymorphism (SSCP) primers. The line chart on the top and bottom panels illustrates the similarity level in percentage (red line) and Shannon’s entropy score (blue line) of each position on the multiple sequence alignment of the *SSU rDNA* gene. The lower panels represent the regions where the forward (SSCP6_F) and reverse (SSCP6_R) primers bind to the *SSU rDNA* gene. The gray arrows represent the direction of the primers.

The MSA of 192 *SSU rDNA* sequences from all 12 genera of dictyostelids was generated. Shannon’s entropy and similarity scores of each MSA position were used to identify the conserved regions in the MSA (Figure 1). A pair of oligonucleotide primers for the SSCP fingerprint method was designed in the region with low Shannon’s entropy scores but high sequence similarity scores. According to the Primer-BLAST results, an approximately 200-bp PCR amplicon could be amplified only for dictyostelids with these candidate primers. However, the primer could generate 978 and 3,865 bp of PCR amplicons from the genomes of *Timema genevievae* (grasshopper) and *Candida intermedia* (ascomycete yeast). Nonetheless, the two non-specific products could be removed from the analysis by size exclusion gel electrophoresis. With these criteria, the primers should be universal and specific for dictyostelids. The forward primer (SSCP6_F) and reverse primer (SSCP6_R) were located at positions 112–132 and 343–360, respectively, in the MSA (Table 1). Both primers were designed to cover a variable region where high Shannon’s entropy and low similarity were presented.

**TABLE 1:** The SSCP primers used in this study.

Primers	Primer sequence (5′–3′)	T_m_* (°C)	Self-dimer** (Δ*G*)	Hairpin** (Δ*G*)	Heterodimer** (Δ*G*)
SSCP6_F	GCA GTA AAT CGG GGC TAA TAC	53.3	−3.61	0.51	−9.75
SSCP6_R	CCC GTT ACA ACC ATG GTA	51.7	−14.2	−0.24	

SSCP, single-strand conformation polymorphism. * Melting temperatures were calculated using OligoAnalyzer^*TM*^ (Integrated DNA Technologies, United States) with Oligo concentration of 0.25 μM and Na^+^ concentration of 50 mM.

** The maximum Gibbs free energies (ΔG) of each secondary structure (the most spontaneous one) are shown.

### Optimization of Single-Strand Conformation Polymorphism Fingerprint Method for Dictyostelids

**FIGURE 2:**
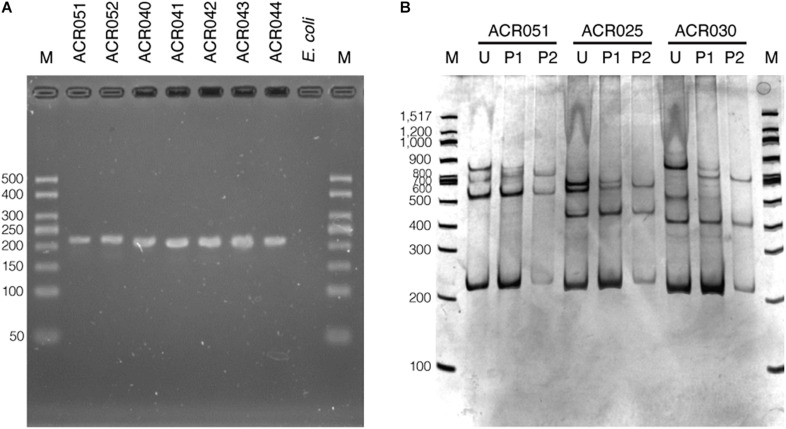
The PCR amplification results using single-strand conformation polymorphism (SSCP) primers and the effects of the amplicon purification methods on the SSCP fingerprint method. The PCR amplicons were amplified using the SSCP primers (SSCP6_F and SSCP6_R) and the genomic DNA templates extracted from each isolate of dictyostelids **(A)**. Lane M represents the 50-bp DNA ladder (Vivantis, Malaysia) followed by seven lanes of PCR amplicons including *Cavenderia* sp. ACR051, *Cavenderia* sp. ACR052, *Dictyostelium* sp. ACR040, *Dictyostelium* sp. ACR041, *Dictyostelium* sp. ACR042, *Dictyostelium* sp. ACR043, and *Dictyostelium* sp. ACR044. The genomic DNA of *Escherichia coli* was used as a negative control. The polyacrylamide gel electrophoresis (PAGE) of SSCP fingerprint of three isolates of dictyostelids was performed, including *Cavenderia* sp. ACR051, *Polysphondylium* sp. ACR025, and *Dictyostelium* sp. ACR030 **(B)**. The SSCP fingerprint from each isolate was generated from the unpurified amplicons (U), directly purified amplicons (P1), and gel purified amplicons (P2). The 9% PAGE was run at 130 V, 4°C for 3 h followed by silver staining. DNA marker (M) in the PAGE was 100-bp DNA ladder (New England Biolabs, United States).

The set of oligonucleotide primers (SSCP6_F and SSCP6_R) successfully amplified an expected ∼200-nt fragment of all 73 dictyostelid Thai isolates without non-specific amplification (Figure 2A). Two purification methods for cleaning up the PCR amplicons were performed before the denaturation step in the SSCP fingerprint method. The first method was direct PCR purification from the crude amplicon (P1). The second method was the gel purification method (P2). The SSCP patterns from the two different DNA purification methods were compared with those of the unpurified PCR products (U). The SSCP fingerprint background of samples with purification methods (P1 and P2) appeared cleaner than those with the unpurified conditions (Figure 2B). Although the direct purification methods (P1) could eliminate the smeared background, they still generated uncertain banding patterns with low intensity. On the other hand, the gel purification method (P2) gave a reproducible SSCP fingerprint with a lower-intensity background compared with the other methods (Figure 2B). Therefore, we performed gel purification for the SSCP fingerprint method for the remainder of this study.

**FIGURE 3:**
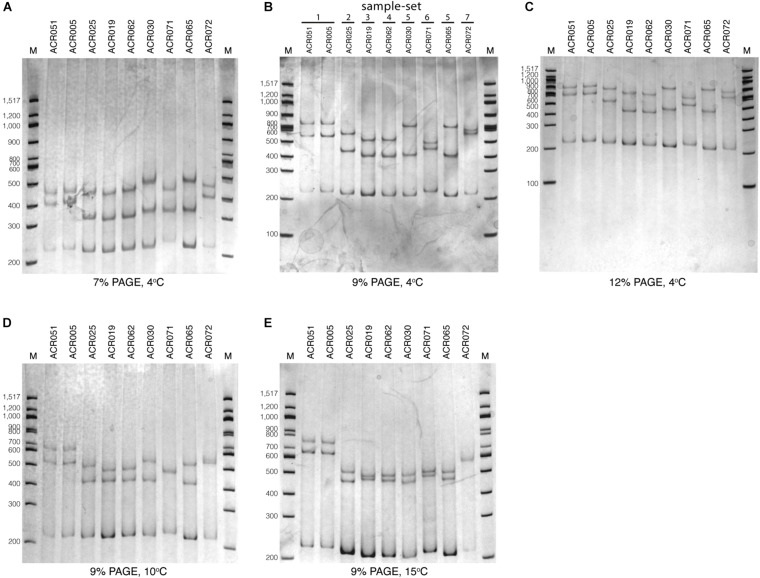
Polyacrylamide gel electrophoresis (PAGE) of the single-strand conformation polymorphism (SSCP) fingerprint under numerous conditions. The SSCP fingerprint method was optimized under several acrylamide concentrations, including 7% **(A)**, 9% **(B)**, and 12% **(C)** and also various temperatures, including 4°C **(B)**, 10°C **(D)**, and 15°C **(E)**. All PAGEs were run at 130 V, 4°C for 3 h, followed by silver staining. Lanes 1–9 are nine dictyostelid samples selected according to the previous phylogenetic tree analysis (Rukseree et al., 2018). The 100-bp DNA ladder (New England Biolabs, United States) was used as the reference marker (lane M).

To optimize the SSCP fingerprint method with reproducibility, ease, and information, we focused on two critical parameters – the concentration of the polyacrylamide gel and the temperature for SSCP run – that had a direct effect on the quality of the SSCP fingerprint. We compared three concentrations of polyacrylamide gel, including 7, 9, and 12%. The results showed that approximately two fingerprint patterns were formed in the 7% polyacrylamide gel (Figure 3A). This concentration apparently could not support band-migration and decreased the discriminatory power of the SSCP fingerprint method. This effect seemed to be solved at the 9 and 12% polyacrylamide gels (Figures 3B,C). In these conditions, the method generated a more significant complexity of banding patterns, which increased the discrimination power of the SSCP method. However, at the concentration of 9% of polyacrylamide, fewer toxic chemicals were used and electrophoresis was less time-consuming. Therefore, we suggested running the SSCP fingerprint of dictyostelids with 9% polyacrylamide gel.

We also compared three different electrophoresis temperatures (4, 10, and 15°C) for the SSCP run. The results showed that cold temperatures, particularly 4°C, could maximize the discrimination power of the SSCP fingerprint method better than higher temperatures (Figure 3B). The higher temperatures (10 and 15°C) greatly affected the migration of the fingerprint bands and also made the fingerprint patterns indistinguishable (Figures 3D,E). So the best temperature for the SSCP method for dictyostelids was 4°C. Based on the above observations, the ideal condition of the SSCP fingerprint method for diversity study of dictyostelids using the *SSU rDNA* gene is the preparation of SSCP amplicons by gel purification and running SSCP fingerprint with 9% polyacrylamide gel at 4°C.

### Profile Diversity of the Thai Isolates of Dictyostelids Based on Single-Strand Conformation Polymorphism Fingerprint

We tested the optimized SSCP fingerprint method on the genetic diversity of 73 dictyostelid Thai isolates from Amnat Charoen province, Thailand. All isolates were divided into seven groups based on the nucleotide similarity of the PCR-SSCP amplicons, approximately 205–220 bp in length (Supplementary Table 2 and Figure 1). The SSCP amplicons of dictyostelid isolates within the same group had exactly 100% nucleotide sequence identity. Dictyostelids in sample-set 1 comprised 17 isolates in the genus *Cavenderia*. The 15 isolates in sample-set 2 belonged to the genus *Polysphondylium*. Sample-set 3 (seven isolates), sample-set 4 (20 isolates), and sample-set 5 (eight isolates) belonged to the genus *Dictyostelium*. Five isolates of sample-set 6 belonged to the genus *Raperostelium*, and a single isolate belonged to the genus *Heterostelium*.

**FIGURE 4:**
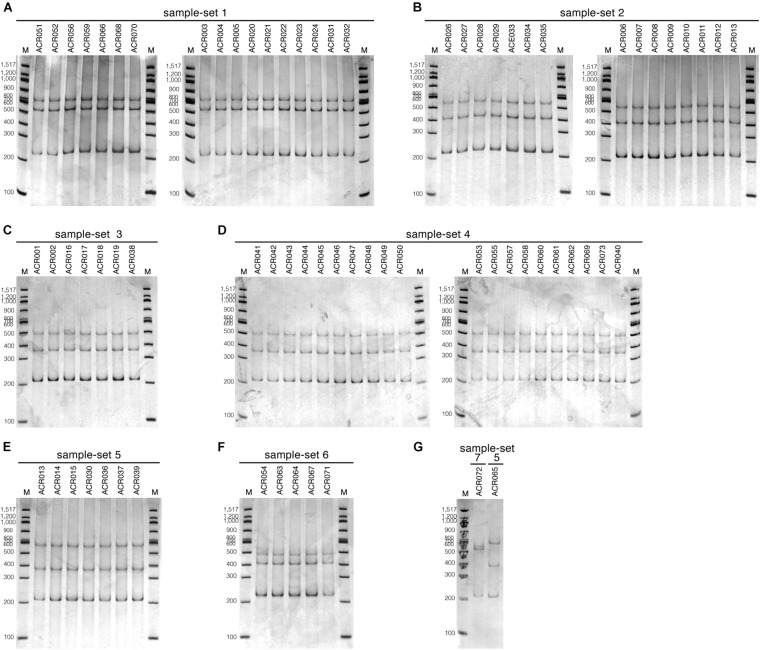
The single-strand conformation polymorphism (SSCP) fingerprint patterns of all 73 dictyostelid Thai isolates used in this study. The SSCP fingerprints were run in 9% polyacrylamide gel electrophoresis (PAGE) under the electrophoretic condition at 130 V, 4°C for 3 h, followed by the silver staining. The 100-bp DNA ladder (New England Biolab, United States) was used as the DNA markers for all PAGE gels (lane M). The SSCP fingerprint pattern of each sample-set is illustrated in separate panels, including sample-set 1 **(A)**, sample-set 2 **(B)**, sample-set 3 **(C)**, sample-set 4 **(D)**, sample-set 5 **(E,G)**, sample-set 6 **(F)**, and sample-set 7 **(G)**.

Analysis of the SSCP fingerprints of the total of 73 dictyostelid Thai isolates demonstrated six different SSCP fingerprint patterns (Figures 3B, 4). All dictyostelid Thai isolates were subjected to the SSCP method more than three times to confirm the reproducibility of the method and the conditions. All the isolates within the same sample-set showed constant unique SSCP fingerprint patterns (Figure 4) except for sample-sets 3 and 4, which gave the same fingerprint despite a single nucleotide difference (Figure 3B).

**FIGURE 5:**
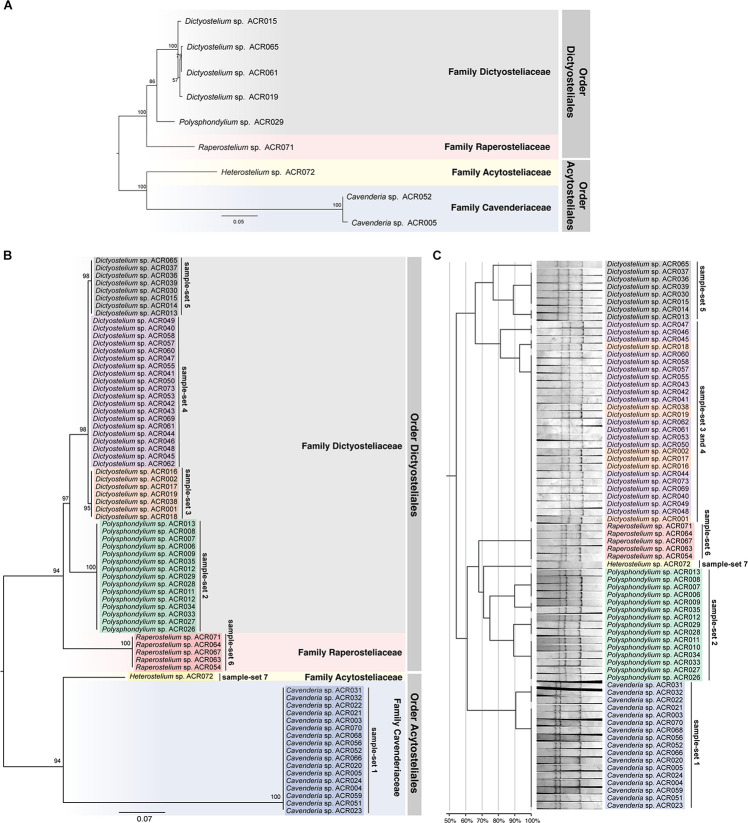
Comparison between the maximum likelihood (ML) phylogenies and the clustering of the single-strand conformation polymorphism (SSCP) fingerprint patterns of all 73 dictyostelid Thai isolates. Molecular phylogeny of the full-length *SSU rDNA* gene sequences was reconstructed using the ML method of nine representative sequences of dictyostelids **(A)**. Each branch on the tree was supported by bootstrap analysis with 1,000 replicates of pseudo-samples. The family and order names of dictyostelids were labeled on the right-hand side of the tree. The scale bar indicates the distance value of each branch. Colored boxes represent the families of dictyostelids. The ML phylogeny was reconstructed from ∼200-bp PCR-SSCP region of 73 dictyostelid Thai isolates **(B)**. Sequences in the different sample-sets are highlighted with different colors, including sample-set 1 (blue), sample-set 2 (green), sample-set 3 (orange), sample-set 4 (purple), sample-set 5 (gray), sample-set 6 (red), and sample-set 7 (yellow). Clustering of the SSCP fingerprint patterns of all 73 isolates of Thai dictyostelids was performed using GelJ software **(C)**. The normalized SSCP fingerprint patterns and taxon labels are illustrated at the right-hand side of the dendrogram. The taxa of the same sample-set are highlighted with the same colors of the ML phylogeny. The scale bar underneath the dendrogram indicates the similarity percentages between each pattern in the dendrogram.

The ML tree was reconstructed from the full-length *SSU rDNA* gene to confirm the genus-level taxonomy of the sample-sets (Figure 5A). The tree was reconstructed from nine full-length *SSU rDNA* gene sequences, which were a representative isolate of each sample-set. All nine isolates illustrated the positions on the tree that resembled the modern taxonomy of dictyostelids in genus-, family-, and order-level taxonomy. Because of the 100% identity of partial sequence at 5′-half of the *SSU rDNA* gene, we assumed that the other isolates in the same sample-set would be identified in the same genus. We also built a molecular phylogeny from the PCR-SSCP amplicon’s sequences that were approximately 200 bp in length (Figure 5B). The PCR-SSCP sequence tree was rooted according to the new classification of dictyostelids. According to Figure 5B, the genus *Cavenderia* (sample-set 1) comprised all isolates that had exactly the same sequences with the isolate *Cavenderia* sp. ACR005 and *Cavenderia* sp. ACR052. That is because the mutations that separated both sequence patterns were presented outside the PCR-SSCP region. The isolate *Heterostelium* sp. ACR072 (sample-set 7) was still the sister taxon of the genus *Cavenderia*. For the genus *Dictyostelium*, the PCR-SSCP sequence phylogeny separated the taxa into three different clades by sample-sets 3–5 (Figure 5B). All isolates that shared a nucleotide sequence in the PCR-SSCP region are exactly the same with *Dictyostelium* sp. ACR019 and *Dictyostelium* sp. ACR061 grouped in sample-sets 3 and 4, respectively. Exceptionally, the isolates that shared 100% sequences in the PCR-SSCP region with *Dictyostelium* sp. ACR015 were grouped together with *Dictyostelium* sp. ACR065 (sample-set 5), because some mutations occurred outside the PCR-SSCP region. All isolates of the genus *Polysphondylium* were the sister clade of all isolates belonging to the genus *Dictyostelium*. Five isolates of the genus *Raperostelium* appeared to be the sister clade of the family Dictyosteliaceae (genus *Dictyostelium* + genus *Polysphondylium*). Obviously, the deep branching topology of the PCR-SSCP phylogeny had the power to represent the full-length *SSU rDNA* gene phylogeny, at least in the genus-level taxonomy.

**FIGURE 6:**
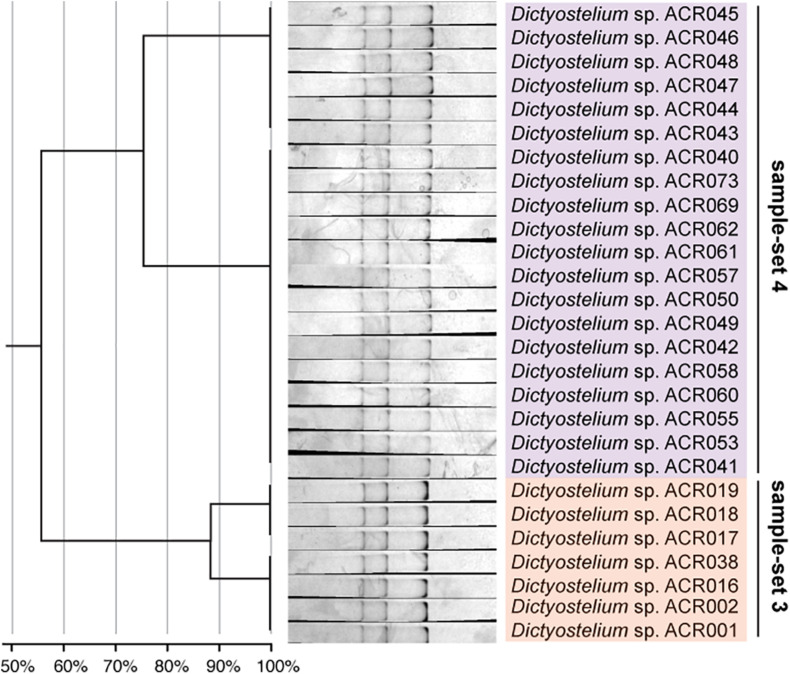
The single-strand conformation polymorphism (SSCP) fingerprint of sample-sets 3 and 4 presented in the separated clusters. The left-hand panel illustrates the dendrogram with the similarity score of the fingerprint patterns. The dendrogram separated the SSCP fingerprint patterns of sample-set 3 (orange) from sample-set 4 (purple).

The dendrogram built from the SSCP fingerprint patterns shows that the isolates of the same sample-set were clustered together, except sample-sets 3 and 4, which were combined into a single cluster (Figure 5C). All isolates of the genus *Dictyostelium* were clustered into a single group and separated from the SSCP fingerprint patterns of the other genera (Figure 5C). The SSCP fingerprint patterns of sample-set 5 appeared to be different from the remaining isolates of the genus *Dictyostelium*. Sample-sets 3 and 4 produced very similar patterns of the SSCP fingerprint. However, fingerprint analysis of sample-sets 3 and 4 alone could discriminate sample-set 3 from sample-set 4 (Figure 6). That was because the migration rate of the third band of sample-set 3 was slightly different from that of sample-set 4. Apart from the genus *Dictyostelium*, the fingerprints of sample-sets 1, 2, 6, and 7 were clustered into the individual group related to the dictyostelids’ genus. The SSCP fingerprint patterns of the genus *Cavenderia* (sample-set 1) were unique and highly homogeneous, with a banding similarity higher than 90%. Although the fingerprint patterns of isolates belonging to the genus *Polysphondylium* (sample-set 2) were slightly less homogenous (a banding similarity of around 75%), all isolates were clustered together. The fingerprint patterns of dictyostelids in the genus *Raperostelium* (sample-set 6) showed the highest homogeneity with 100% banding similarity support. *Heterostelium* sp. ACR072, a single isolate of sample-set 7, also illustrated the unique fingerprint pattern. That was because the first and second bands migrated at almost the same rate (the bands were located close to each other).

### Fingerprint Patterns of the Genus *Dictyostelium*

Unlike the fingerprint patterns of most genera of dictyostelids that produced a single pattern per genus, the genus *Dictyostelium* produced two different SSCP fingerprint patterns. One was the cluster of sample-set 5, which was significantly separated from another pattern. Another was the cluster of the mixture between sample-sets 3 and 4 (Figure 5C). Comparison of the banding similarity scores of the two fingerprint patterns showed a translated band into 65% banding similarity. Interestingly, the phylogenetic analysis of the PCR-SSCP amplicons differentiated those three sample-sets (sample-sets 3–5) with 97% bootstrap support (Figure 5B), whereas the SSCP pattern of sample-set 3 was relatively similar to that of sample-set 4 with a banding similarity of more than 80% (Figure 5C). To investigate the fingerprint pattern variation between sample-sets 3 and 4, we reanalyzed the fingerprint patterns using only 27 isolates of sample-sets 3 and 4 to avoid the heterogeneity effects of other fingerprint patterns that would interfere with the system. The results showed that without the larger heterogeneity patterns, sample-set 3 was clearly separated from sample-set 4 (Figure 6).

**FIGURE 7:**
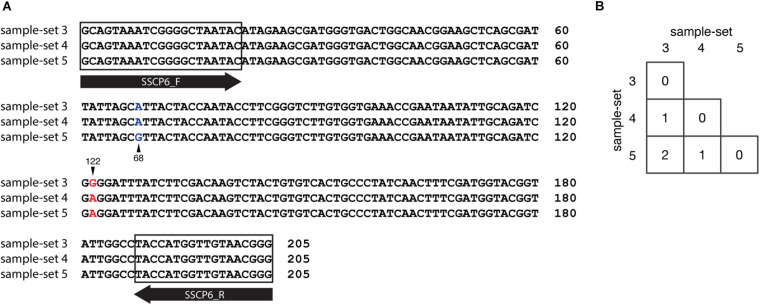
Nucleotide substitution variations among the single-strand conformation polymorphism (SSCP) regions of sample-sets 3–5. Multiple sequence alignment of 205 nucleotides of SSCP sequences of sample-sets 3–5 was generated. **(A)** Nucleotide substitutions at positions 68 (column with blue text) and 122 (column with red text). The sample-set is presented on the left-hand side of the alignment, whereas the nucleotide position is shown on the right. Primer binding regions and direction are shown in the gray boxes and open arrows, respectively. The number of nucleotide substitutions between each nucleotide sequence pair is shown in the matrix **(B)**.

We also compared the *SSU rDNA* gene sequences among the three sample-sets (sample-set 3 to sample-set 5). The MSA of 205 nucleotides of the three sample-sets identified two nucleotide substitutions at positions 68 and 122 in the alignment (Figure 7A). The nucleotide substitution at position 68 was changed from adenine (A) in sample-sets 3 and 4 to guanine (G) in sample-set 5 (A68G). The nucleotide substitution at position 122 was changed from guanine (G) in sample-set 3 to adenine (A) in sample-sets 4 and 5 (G122A). Surprisingly, the nucleotide sequences of sample-sets 3 and 4 differed by one substitution, and sample-sets 4 and 5 differed by another substitution (Figure 7B). However, the SSCP fingerprint patterns of sample-set 5 were significantly different from the others.

**FIGURE 8:**
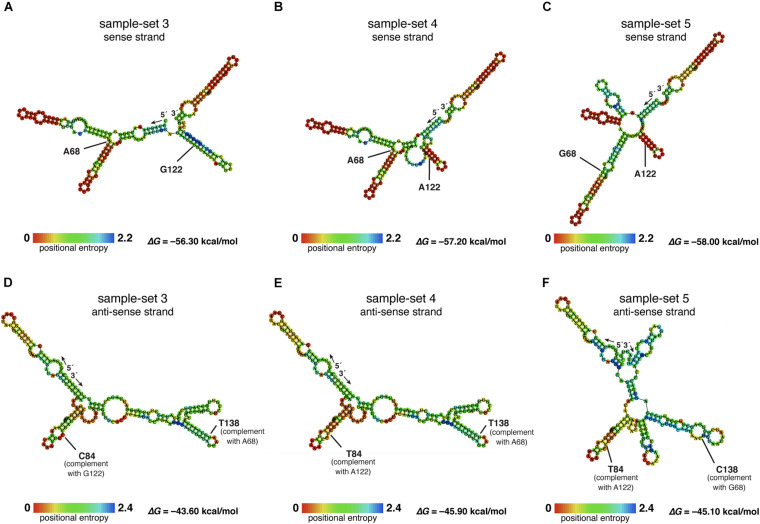
Putative RNA secondary structure of the PCR–single-strand conformation polymorphism (SSCP) amplicons of sample-sets 3–5. The putative RNA folding structures based on the minimum free energy (△*G*) of the PCR-SSCP amplicons of sample-set 3 **(A,D)**, sample-set 4 **(B,E)**, and sample-set 5 **(C,F)** were generated by ViennaRNA Package 2.0 (Lorenz et al., 2011). The top panels are the putative structures of the sense strand of each sample-set, whereas the bottom panels are the putative structures of the anti-sense strand of each sample-set. Ribonucleotide residue at substitution sites 68 and 122 of the three sense strands are labeled with ribonucleotide abbreviation adjacent to the position, as A68, G68, G122, and A122 **(A–C)**. Positions 138 and 84 of the anti-sense strand, which corresponded to positions 69 and 122 of the sense strand, respectively, are labeled as C84, T84, T138, and C138. The free energy (△*G*) values of the most stable RNA secondary structure of each sample-set are presented at the lower-right corner. The rainbow bars at the lower-left corner of each structure demonstrate positional entropy of ribonucleotide subunits.

The nucleotide sequences of sample-sets 3, 4, and 5 were further subjected to the secondary structure prediction of the single-strand conformations based on the MFE (△*G*) (Figure 8). The results showed that the effect of the substitution strongly influenced the single-strand conformation of sample-set 5 in both sense and anti-sense strands of the single-strand PCR-SSCP amplicons. This made the single-strand conformation of sample-set 5 differed from that of sample-sets 3 and 4 (Figures 8C,F). Emphasizing sample-sets 3 and 4, the substitution at position 122 (G122A) seemed to be less affected on the folding conformation of that sample-set.

## Discussion

We designed a new set of universal SSCP primers to amplify about 200 bp of the *SSU rDNA* gene of dictyostelids that could be used to generate an SSCP fingerprint useful for genetic diversity study among dictyostelids (Figure 1 and Table 1). The primers successfully amplified the *SSU rDNA* gene, and we also found that the gel purification method reduced the inconsistency band and reduced the fingerprint background (Figure 2). We optimized the SSCP conditions to enhance the discriminatory power and reproducibility of the SSCP fingerprint for dictyostelids (Figure 3). The SSCP fingerprint method was tested with a set of 73 dictyostelid Thai isolates (Figure 4). Nine representative sequences of all 73 isolates underwent phylogenetic analysis using ML method for confirming their genus-level taxonomy (Figure 5A). A molecular phylogeny was reconstructed from the nucleotide sequences of the PCR-SSCP region to test phylogenetic power of this region (Figure 5B). Although the tree from PCR-SSCP region had less phylogenetic power than the full-length *SSU rDNA* gene, the deep branches were correlated to the genus-level taxonomy. The dendrogram built from the fingerprint patterns clustered dictyostelids by their genus (Figure 5C). Unexpectedly, the analysis results showed that sample-sets 3 and 4 were mixed in the same cluster. However, after we thoroughly examined the results by focusing on only sample-sets 3 and 4, all isolates of sample-set 3 were separated from sample-set 4 on the basis of the slight difference of the third band position (Figure 6). Even though sample-sets 3 and 4 differed by one nucleotide substitution (Figure 7), the substitution could not significantly change the single-strand conformation (Figure 8).

The designation of primers is the first key to generate informative SSCP fingerprint patterns. The selected PCR-SSCP region covered the V2 region, which is one of the hypervariable regions of the *SSU rDNA* gene. The set of oligonucleotide primers and the optimized SSCP fingerprint method successfully generated the fingerprint patterns that were correlated with the amplicon’s sequences (Figure 5). Interestingly, the SSCP fingerprint method generated three unique patterns specific to the *Dictyostelium* genus sequences (Figure 5C). The fingerprint patterns of each genus of dictyostelids were different. It might be inferred that our optimized SSCP fingerprint method has the potential for dictyostelid identification, at least at the genus-level taxonomy.

Undoubtedly, the sequence of full-length *SSU rDNA* gene has more power than the PCR-SSCP region within the *SSU rDNA* gene and also has more power than any fingerprint method. Moreover, phylogenetic tree reconstruction using ML method strongly relied on a nucleotide substitution model (Stamatakis, 2014), whereas the dendrogram was built from the similarity of fingerprint patterns (Heras et al., 2015) without implementation of any evolutionary model. Therefore, it is not surprising that the deep branching pattern of the dendrogram built from the SSCP fingerprint patterns was incongruent with the sequence phylogenies (Figure 5). Fortunately, the tree built from PCR-SSCP sequences (Figure 5B) illustrated the deep branching topology as the full-length *SSU rDNA* gene phylogeny (Figure 5A). This is because the 5′-end of the *SSU rDNA* gene is known to contain evolutionarily informative signature sequences (Baldauf et al., 2018; Rukseree et al., 2018; Sheikh et al., 2018). However, considering the ML trees reconstructed from the PCR-SSCP amplicons, the partial *SSU rRNA* (Figures 5A,B), and the *SSU rDNA* gene phylogenies (Baldauf et al., 2018; Rukseree et al., 2018; Sheikh et al., 2018), short nucleotide region in the PCR-SSCP region has some phylogenetically informative signals for identification of the dictyostelids at the genus-level taxonomy.

Although our sample-sets comprised one unique *SSU rDNA* gene for any genus, at least three sequences differed in the sample-set from the genus *Dictyostelium*. The diversity of dictyostelids in the genus *Dictyostelium* appeared more diverse than that of the other four genera of dictyostelids in our sample-sets (Rukseree et al., 2018). The species of those Thai isolates in the genus *Dictyostelium* were still not identified, but the three different sequences generated three fingerprint patterns. Sample-set 5 shared about 65% similarity in the fingerprint patterns with the remaining isolates of the genus *Dictyostelium*. The result seems to be conflicted with the sequence identities between sample-set 5 and the other sample-sets within the same genus that differed with only one or two nucleotide substitutions (Figure 7). However, the secondary structures of the PCR-SSCP amplicons of those three sample-sets of the genus *Dictyostelium* illustrated that the structures of sample-set 5 look significantly different from the other two structures. That is the reason that the SSCP fingerprint pattern of sample-sets 3 and 4 resembled each other.

Theoretically, a short fragment of about 200 nucleotides in length is more suitable for detecting a mutation using the SSCP fingerprint method than the longer ones. It has been claimed that the SSCP fingerprint method has the potential to separate the ssDNA conformation from one substitution mutation (Hayashi, 1991, 1992; Hayashi and Yandell, 1993). However, the SSCP fingerprint method could not generate significant differences in the conformations for sample-sets 3 and 4. Furthermore, the primers we used in the study have generated the SSCP fingerprint patterns with three bands in all 73 isolates, which were less than those of the other studies in the other group of organisms (Barroso et al., 1998; Rehbein et al., 1999). This is because the nature of the *SSU rDNA* gene, which was selected as the target for the SSCP fingerprint method, would automatically form a secondary structure for its function (Olsen et al., 1983). The structure is very stable and easily formed with very low Gibbs free energy (Mathews and Turner, 2006). We hypothesized that the low Gibbs free energy (△*G* less than -56 kcal/mol) accommodates stable conformations, which contributed to the reproducibility of the three bands. Moreover, the nucleotide substitution at position 68 had affected the secondary structure of the nucleotides by extension of the stem region in the secondary structure (Figure 8C), while the mutation at position 112 showed a negligible effect on conformation change. However, the mechanism of how a substitution affects the conformation or how the conformation affects the migration during electrophoresis is unknown (Chrambach and Rodbard, 1971; Orita et al., 1989).

In terms of molecular systematics, there were no fingerprint methods more powerful than sequence phylogeny. Nevertheless, the advantages of the SSCP fingerprint method are cheaper, faster, easier to perform in most molecular biology laboratories using only basic laboratory equipment, and less time-consuming for data analysis than phylogenetic analysis using the *SSU rDNA* gene (Sunnucks et al., 2000). The *SSU rDNA* gene phylogeny is still a standard for species identification and evolutionary study of dictyostelids. Thus, the major use of our proposed method is to save costs for screening of the redundant clones in a massive survey of dictyostelids. Fortunately, with thorough primers design and optimization, the method could be used for molecular identification of dictyostelids at least in the genus-level taxonomy. In addition, the DNA fingerprint methods were used as tools for studying genetic diversity for decades. This SSCP method could be an alternative tool for measurement of genetic diversity of dictyostelids.

## Conclusion

In this study, we proposed an optimized method for the generation of the SSCP fingerprint patterns. The method is cheaper, faster, more time-saving, and more convenient for massive samples than the *SSU rDNA* gene phylogeny. It also has discriminatory power in at least the genus-level taxonomy, an alternative method for genus identification. However, this method would be more powerful if one implements more than one set of primers to increase the band complexity, which might increase the chance to explore species-specific markers.

## Data Availability Statement

The original contributions presented in the study are included in the article/Supplementary Material, further inquiries can be directed to the corresponding author.

## Author Contributions

PA conceived the study. AU, KS, and PhP conducted the experiments. PhP analyzed the data. PrP, KR, KA, SB, and SS advised in both experimental and analytical sessions. PhP and PA wrote the manuscript. All authors read and approved the manuscript.

## Conflict of Interest

The authors declare that the research was conducted in the absence of any commercial or financial relationships that could be construed as a potential conflict of interest.

## Publisher’s Note

All claims expressed in this article are solely those of the authors and do not necessarily represent those of their affiliated organizations, or those of the publisher, the editors and the reviewers. Any product that may be evaluated in this article, or claim that may be made by its manufacturer, is not guaranteed or endorsed by the publisher.

## Footnotes

1
https://www.idtdna.com/calc/analyzer

2
http://rna.tbi.univie.ac.at/cgi-bin/RNAWebSuite/RNAfold.cgi

## Supplementary Material

Supplementary Table 1 List of the accession numbers of dictyostelids in the multiple sequence alignment (MSA) for designing of the PCR-SSCP primers.

Supplementary Table 2 List of the total 73 dictyostelid isolates in this study (Rukseree et al., 2018).
