# Supplementary material for: Single-Strand Conformation Polymorphism Fingerprint Method for Dictyostelids
Source: Front Microbiol. 2021 Aug 27;12:708685. doi: 10.3389/fmicb.2021.708685 (PMC8431811; doi:10.3389/fmicb.2021.708685)
Supplement: Supplementary Table 1 — List of the accession numbers of dictyostelids in the multiple sequence alignment (MSA) for designing of the PCR-SSCP primers. [file Data_Sheet_1.PDF]

Table S1 List of the total 192 dictyostelid species for the SSCP primer design

Genus	Species	Strain	Accession no.
Acytostelium	*A. longisorophorum*		AM168109
	A. subglobosum	LB1	AM168110
	A. leptosomum	FG12	AM168111
	A. serpentarium		AM168113
	A. digitatum	OH517	AM168114
	A. anastomosans		AM168115
	A. amazonicum	landolt	HQ141510
	A. amazonicum	HN1B1	HQ141511
	A. leptosomum	212rjb	HQ141512
	A. magnisorum	08A	HQ141513
	A. singulare	FDIB	HQ141514
Cavenderia	*Cavenderia sp.*	ACR005	MG754877
	Cavenderia sp.	ACR052	MG755024
	Ca. multistipes	UK26b	AM168070
	Ca. granulophora	CHII4	AM168072
	Ca. antartica	NZ43B	AM168080
	Ca. stellata	SAB7B	AM168081
	Ca. aureostipes	YA6	AM168083
	Ca. bifurcata	UK5	AM168084
	Ca. exigua	TNSC199	AM168085
	Ca. fasciculata	SmokOW9A	AM168086
	Ca. fasciculata	SH3	AM168087
	Ca. medusoides	OH592	AM168088
	Ca. mexicana	Mex TF4B1	AM168089
	Ca. microspora	TNSC38	AM168090
	Ca. parvispora		AM168091
	Ca. deminutiva	MexM19A	AM168092
	Ca. delicata		AM168093
	Ca. fasciculoidea		GQ496157
	Ca. subdiscoidea	TH1A	HQ141515
	Cavenderia sp.	TAS30A	HQ141516
	Cavenderia sp.	TH18B	HQ141517
	Ca. pseudoaureostipes	TH39A	HQ141518
	Ca. macrocarpa	MGE2	HQ141519
	Ca. amphispora		HQ141521
	Ca. myxobasis	NT2A	HQ141522
	Ca. bhumiboliana	TH11CX	HQ141523
	Ca. aureostipes	B15A	KF662199
	Ca. aureostipes	OH396	KF662201
	Ca. aureostipes var.helvetium	HM592	KF662214
	Ca. aureostabilis	TH10B	MH745571
	Ca. protodigitata	TH18BA	MH745572
Coremiostelium	*Co. polycephalum*	MY1	AM168056
	Co. polycephalum	SS3B	HQ141488
	Co. polycephalum	2132	HQ141489

Table S1 List of the total 192 dictyostelid species for the SSCP primer design (Cont'd)

Genus	Species	Strain	Accession no.
Coremiostelium	*Co. polycephalum*	1675	HQ141490
Dictyostelium	*Dictyostelium sp.*	ACR015	MG754987
	Dictyostelium sp.	ACR019	MG754991
	Dictyostelium sp.	ACR061	MG755033
	Dictyostelium sp.	ACR065	MG755037
	D. auroecephalum	TNSC180	AM167876
	D. aureum	SL1	AM168028
	D. brefeldianum	TNSC115	AM168030
	D. brunneum	WS700	AM168031
	D. capitatum	91HO50	AM168032
	D. citrinum	OH494	AM168033
	D. clavatum	TNSC189	AM168034
	D. clavatum	TNSC220	AM168035
	D. crassicaule	93HO33	AM168037
	D. dimigraformum	AR5b	AM168038
	D. discoideum	V34	AM168039
	D. firmibasis	TNSC14	AM168041
	D. giganteum	WS589	AM168042
	D. implicatum	93HO1	AM168043
	D. intermedium	PJ11	AM168044
	D. longosporum	TNSC109	AM168048
	D. macrocephalum	B33	AM168049
	D. medium	TNSC205	AM168050
	D. mucoroides	TNSC114	AM168053
	D. mucoroides	S28b	AM168054
	D. mucoroides	VSFOII1	AM168055
	D. pseudobrefeldianum	91HO8	AM168059
	D. purpureum	C143	AM168060
	D. purpureum	WS321	AM168061
	D. robustum	TNSC219	AM168064
	D. rosarium	M45	AM168065
	D. septentrionalis	IY49	AM168066
	D. septentrionalis	AK2	AM168067
	D. sphaerocephalum	GR11	AM168068
	D. discoideum	NC4	AM168071
	D. purpureum		AY040335
	D. discoideum	AX4	G0295647
	D. purpureum		DQ340386
	D. purpureum	QSpu4	FJ424826
	D. purpureum	QSpu36	FJ424828
	D. purpureum	QSpu1	FJ424829
	D. purpureum	QSpu23	FJ424832
	D. purpureum	QSpu28	FJ424836
	D. purpureum	QSpu2	FJ424839
	D. valdivianum	Vald 3C	GQ496155

Table S1 List of the total 192 dictyostelid species for the SSCP primer design (Cont'd)

Genus	Species	Strain	Accession no.
Dictyostelium	*D. austroandinum*	Blest 5	GQ496158
	D. chordatum	Moreno 7	GQ496159
	D. gargantuum	Puelo 1	GQ496161
	D. leptosomopsis	Araucaria1	HM159992
	D. ammophilum	KBK4A	HQ141478
	D. quercibrachium	NZ201B	HQ141479
	D. leptosomum	NZN49A	HQ141480
	D. purpureum		HQ141481
	D. mucoroides	Sweden20	HQ141482
	Dictyostelium sp.	Laos1	HQ141483
	Dictyostelium sp.	Laos5	HQ141484
	D. barbibulus	Sweden4R	JX173878
Hagiwaraea	*Ha. coeruleostipes*	CR_LC53B	AM168036
	Ha. lavandula	B15	AM168047
	Ha. vinaceofusca	CC4	AM168062
	Ha. rhizopodium	AusKY-4	AM168063
	Ha. radiculata	ML5A	HQ141494
Heterostelium	*Heterostelium sp.*	ACR072	MG755044
	He. gloeosporum	TCK52	AM168074
	He. oculare	B4B	AM168079
	He. anisocaule	NZ47B	AM168096
	He. asymetricum	OH567	AM168097
	He. colligatum	OH538	AM168098
	He. equisetoides	B7JB	AM168099
	He. filamentosum	SU1	AM168100
	He. luridum	LR2	AM168101
	He. arachnoideum	YA1	AM168102
	He. pallidum	TNSC98	AM168103
	He. album	PN500	AM168104
	He. tenuissimum	TNSC97	AM168105
	He. tikalense	OH595	AM168106
	He. pseudocandidum	TNSC91	AM168107
	He. candidum		AY040337
	He. tenuissimum		AY040339
	He. pallidum	PHU8	EU004605
	Heterostelium sp.	Laos3	HQ141496
	He. oculare		HQ141497
	He. candidum	bsb6b	HQ141498
	He. boreale	BSB10A	HQ141499
	He. flexuosum	AU4B	HQ141500
	He. rotatum	QC2C	HQ141501
	He. granulosum	MF5A	HQ141502
	He. asymetricum	HN20C	HQ141503
	Heterostelium sp.	TH12A	HQ141504
	He. colligatum	HN13C1	HQ141505

Table S1 List of the total 192 dictyostelid species for the SSCP primer design (Cont'd)

Genus	Species	Strain	Accession no.
Heterostelium	*He. multicystogenum*	AS2	HQ141506
	He. stolonicoideum	K12A	HQ141507
	He. australicum	NB1AP	HQ141508
	He. tikalense	HN1C1	HQ141509
	He. pseudoplasmodiomagnum		KP167472
	He. unguliferum		KP167473
	He. pseudocolligatum		KP167474
	He. plurimicrocystogenum		KP167475
	He. racemiferum		KP167476
	He. lapidosum		KP167477
	He. violaceotypum		KP167478
	He. cumulocystum		KP167479
	He. ampliverticillatum		KP167480
	He. migratissimum		KP167481
	He. pseudoplasmodiofascium		KP167482
	He. parvimigratum		KP167483
Polysphondylium	*Polysphondylium sp.*	ACR029	MG755001
	P. laterosorum	AE4	AM168046
	P. violaceum	P6	AM168108
	P. patagonicum	H-H 1	GQ496156
	Polysphondylium sp.	Laos4	HQ141485
	P. violaceum	209	HQ141486
	Polysphondylium sp.	Tibet10A	HQ141487
	P. fuscans	Sweden11D	JX173877
Raperostelium	*Raperostelium sp.*	ACR071	MG755043
	Ra. australe	NZ80B	AM168029
	Ra. minutum		AM168051
	Ra. monochasioides	HAG653	AM168052
	Ra. potamoides	FP1A	AM168069
	Ra. tenue	PR4	AM168075
	Ra. tenue	Pan52	AM168076
	Ra. gracile	KP51	AM168078
	Ra. tenue	PJ6	AM168094
	Raperostelium sp.	MR2008	EU672875
	Raperostelium sp.	TH14B	HQ141491
	Raperostelium sp.	TH8C	HQ141492
	Ra. ohioense	Okla4C	HQ141493
	Ra. ibericum	214rjb	HQ141495
	Ra. reciprocatum		JF892718
	Raperostelium sp.	MR2011f	JF892719
	Ra. capillare		JF892721
	Raperostelium sp.	MR2011k	JF892723
	Ra. filiforme		JF892724
	Ra. reciprocatum	38A	Sheikh et al., 2018*
	Raperostelium sp.	PJ2C	Sheikh et al., 2018*

Table S1 List of the total 192 dictyostelid species for the SSCP primer design (Cont'd)

Genus	Species	Strain	Accession no.
Raperostelium	*Ra. transitium*	OH601	Sheikh et al., 2018*
Rostrostelium	*Ro. ellipticum*	AE2	AM168112
Speleostelium	*Sp. caveatum*	WS695	AM168077
Synthelium	*Sy. polycarpum*	VE1b	AM168057
	Sy. polycarpum	OhioWILDS	AM168058
Tieghemostelium	*T. lacteum*		AM168045
	T. menora	M1	AM168073
	T. angelicum		JF892716
	T. montium		JF892717
	T. simplex		JF892720
	T. dumosum		JF892722
	T. unicornutum		JF892725

* = The nucleotide sequences were downloaded from the supplementary material of the article.
