# Supplementary material for: Single-Strand Conformation Polymorphism Fingerprint Method for Dictyostelids
Source: Front Microbiol. 2021 Aug 27;12:708685. doi: 10.3389/fmicb.2021.708685 (PMC8431811; doi:10.3389/fmicb.2021.708685)
Supplement: Supplementary Table 2 — List of the total 73 dictyostelid isolates in this study (Rukseree et al., 2018). [file Data_Sheet_2.PDF]

Table S2 List of the total 73 dictyostelid isolates in this study (Rukseree et al., 2018).

Sample-set	Genus	Isolate	Accession no.
1	*Cavenderia*	ACR003	MG754975
		ACR004	MG754976
		ACR005	MG754977
		ACR020	MG754992
		ACR021	MG754993
		ACR022	MG754994
		ACR023	MG754995
		ACR024	MG754996
		ACR031	MG755003
		ACR032	MG755004
		ACR051	MG755023
		ACR052	MG755024
		ACR056	MG755028
		ACR059	MG755031
		ACR066	MG755038
		ACR068	MG755040
		ACR070	MG755042
2	*Polysphondylium*	ACR006	MG754978
		ACR007	MG754979
		ACR008	MG754980
		ACR009	MG754981
		ACR010	MG754982
		ACR011	MG754983
		ACR012	MG754984
		ACR025	MG754997
		ACR026	MG754998
		ACR027	MG754999
		ACR028	MG755500
		ACR029	MG755001
		ACR033	MG755005
		ACR034	MG755006
		ACR035	MG755007
3	*Dictyostelium*	ACR001	MG754973
		ACR002	MG754974
		ACR016	MG754988
		ACR017	MG754989
		ACR018	MG754990
		ACR019	MG754991
		ACR038	MG755010
4	*Dictyostelium*	ACR040	MG755012
		ACR041	MG755013
		ACR042	MG755014
		ACR043	MG755015
		ACR044	MG755016
		ACR045	MG755017
		ACR046	MG755018
		ACR047	MG755019
		ACR048	MG755020
		ACR049	MG755021

Table S2 List of the total 73 dictyostelid isolates in this study (Cont'd) (Rukseree et al., 2018).

Sample-set	Genus	Isolate	Accession no.
		ACR050	MG755022
		ACR053	MG755025
		ACR055	MG755027
		ACR057	MG755029
		ACR058	MG755030
		ACR060	MG755032
		ACR061	MG755033
		ACR062	MG755034
		ACR069	MG755041
		ACR073	MG755045
5	*Dictyostelium*	ACR013	MG754985
		ACR014	MG754986
		ACR015	MG754987
		ACR030	MG755002
		ACR036	MG755008
		ACR037	MG755009
		ACR039	MG755011
		ACR065	MG755032
6	*Raperostelium*	ACR054	MG755026
		ACR063	MG755035
		ACR064	MG755036
		ACR067	MG755039
		ACR071	MG755043
7	*Heterostelium*	ACR072	MG755044
